# Supplementary material for: Selective filling of n-hexane in a tight nanopore
Source: Nat Commun. 2021 Jan 12;12:310. doi: 10.1038/s41467-020-20587-1 (PMC7804426; doi:10.1038/s41467-020-20587-1)
Supplement: Supplementary file 1 — Supplementary Information [file 41467_2020_20587_MOESM1_ESM.pdf]

Supplementary Material

Selective Filling of n-Hexane in a Tight Nanopore

Haoran Qu[1], Archith Rayabharam[2], Xiaojian Wu[1], Peng Wang[1], Yunfeng Li[1], Jeffrey Fagan[3], Narayana R. Aluru[2], YuHuang Wang[1,4*]

1 Department of Chemistry and Biochemistry, University of Maryland, College Park, MD 20742, United States
2 Department of Mechanical Science and Engineering, University of Illinois at Urbana-Champaign, Urbana, IL 61801, United States
3 Materials Science and Engineering Division, National Institute of Standards and Technology, Gaithersburg, MD 20899 United States
4 Maryland NanoCenter, University of Maryland, College Park, MD 20742 United States

*Correspondence should be addressed to Y.H.W. (yhw@umd.edu)

Supplementary Note 1: Estimation of the numbers of filled n-hexane molecules in (6,5)-SWCNTs based on hyperspectral imaging

To estimate the number of filled n-hexane molecules, we have to consider the diffraction limit. The pixel size (≈ 160 nm) is smaller than the diffraction limit, which stands at ≈ 580 nm in our case (calculated form $\frac{\lambda}{2\mathrm{NA}}$, where λ is the wavelength of the 980 nm emission from (6,5)-SWCNT, and NA = 0.85 is the numerical aperture of the objective used). For this work we used long nanotubes (> 3 μm) to achieve sufficient spatial resolution of PL imaging. It is not possible to completely deconvolute the complexity formed by so many emitting nanotube segments when they are partially filled. However, we may estimate the number of n-hexane molecules in each pixel based on the assumption that strain and PL shift correlation is linear.

In the case of n-hexane filling, the major cause of the photoluminescence (PL) shift is the strain, which is described by Yang's model[1] and other literature reports[2]. The PL shift (ΔE) is observed to be linearly proportional to the strain[3]. Due to the single-file packing, we can consider the system as one dimensional such that the strain is linearly correlated with the number of filled molecules. If this assumption holds, the strain and resulting PL shift is then directly proportional to the number of n-hexane molecules within the nanotube pore.

By considering a linear scaling of the PL peak shift for the empty (0 %) up to the fully filled (100 %) nanotubes, we can obtain the percentage (P) of the nanotube filled with n-hexane from the E_{11} peak wavelength by the following relation:

$$P = kE_{11} - b \qquad (1)$$

where E_{11} is the PL emission peak wavelength in nm, k and b are two constants due to the linear relationship of strain and resulting PL. We then substitute (E_{11} = 982, P = 0%) and (E_{11} = 998, P = 100%) into the equation to obtain k (= 0.0625) and b (= 61.375) for this case.When one n-hexane fills inside a nanotube segment, the length of this segment is roughly equal to the length of the stretched n-hexane (1.14 nm, based on the atom center-to-center length of n-hexane, 0.90 nm, plus the van der Waal radii of the two hydrogen atoms, 2 x 0.12 nm = 0.24 nm). Based on these, the numbers of n-hexane can be derived. For example, for pixels that have an E_{11} peak of 990 nm in Figure 2b, we can calculate the percent filling of the nanotube is 50% n-hexane by plugging in E_{11} = 990 nm. We can further derive that on average there are ≈ 0.43 hexane molecules per nanometer length of (6,5)-SWCNT.

We note that the peak positions (Figure 2a,b) used in the above calculations are from fitting the spectra with a Gaussian profile. The spectra are best fit with a Voigt distribution function which is a result of the convolution of a Gaussian profile (inhomogeneous broadening) and a Lorentzian profile (homogeneous broadening).[4] Although the linewidths are different, all these functions give the same peak positions in our case, as shown in Supplementary Figure 2. Because the Voigt function is computationally more expensive to implement to fit the cubes from hyperspectral imaging, we have used a Gaussian function for the spectral fitting when only the peak position is needed, including Figure 2a, 2b and Supplementary Figure 3. In all the other cases, a Voigt function is used.

Supplementary Note 2: Estimation of the percentage of the pore volume of the tubes filled with n-hexane.

In Figure 4, we show that the n-hexane can be removed from the mixture by (6,5)-SWCNTs. To estimate the filled pore volume, we assume all the SWCNTs are (6,5)-SWCNTs, though we note the nanotube raw material is synthesized as a mixture of different chiralities, with (6,5)-SWCNTs being the major component. We also considered the SWCNT purity (≈ 77.1 mass % based on the manufacturer's product specs).

Adsorption of n-hexane should result in the n-hexane molecules packing single-file inside the (6,5)-SWCNTs due to the size limitation. Therefore, we can estimate the percentage of the pore volume filled with n-hexane based on the length of the molecule and that of the SWCNTs. Since only stretched n-hexane can enter (6,5)-SWCNTs, the molecular configuration that fits the nanotube has a length of 1.14 nm. Therefore, we can estimate when 100 % filled, these nanotubes can densely encapsulate at least 2.07 μmol/mL, 3.68 μmol/mL, 7.52 μmol/mL of n-hexane inside 2.51 mg/mL, 4.47 mg/mL, 9.13 mg/mL (6,5)-SWCNTs, respectively. The actual amount of n-hexane removed was experimentally determined to be 1.56 μmol/mL, 2.63 μmol/mL, 3.92 μmol/mL, respectively (Figure 4). From the ratios, we obtained the percentage of the filled space to range from 52% to 82 % for the experiments performed here. These data suggest that the molecules are loosely packed inside the nanotube pore.

Supplementary Note 3: Raman spectroscopy

We conducted Raman spectroscopy to further investigate the effect of SWCNT strain. The D peak of SWCNTs, which originate from the structural disorder, are commonly located at ≈ 1355 cm^{-1}, while G peaks are at ≈ 1585 cm^{-1} and represent the ordered graphitic structure. Supplementary Figure1a shows the G and D peaks of the Raman spectra from the end-opened and end-capped nanotubes. After the end opening process, the D/G ratio slightly increases from 0.021 to 0.032. This low increase of disorder suggests the amount of nanotube oxidation during the opening process was small. Furthermore, such oxidative defects are known to quench the SWCNT photoluminescence (PL).[5] However, we observed bright PL from the nanotubes (Figure 2 and Supplementary Figure 4), indicating the effect of changing PL properties in terms of peak wavelength and intensity caused by these defects was negligible.

Supplementary Figure 1b shows the G peaks of n-hexane- and water-filled (6,5)-enriched SWCNTs. Note: Due to the ensemble averaging, the G-band and D band signal may be convoluted with contributions from other minor species, such as (8,3) and (7,5). The downshift (3 cm^{-1}) of the G peak of the n-hexane-filled SWCNTs compared to the water-filled nanotubes indicates endohedral encapsulation of n-hexane creates radial expansive strain on the nanotube sidewalls, which is consistent with ref.[6]. The radial breathing mode (RBM) o peak of the n-hexane-filled (6,5)-SWCNTs is ≈313 cm^{-1}, which is upshifted 4 cm^{-1} compared with the water-filled control (Supplementary Figure1c).

Supplementary Note 4: Grid size used in simulation

To have more confidence on the inferences we might have made from the 1x1x4 grid, we performed additional simulations to compare the energies obtained from using a finer grid. We find that the energies obtained from the 4x4x1, 2x2x2 and 4x4x4 grid differ from the values obtained from the 1x1x4 by around 0.005 eV (Supplementary Table 4). These finer grids have higher number of k-points, making them computationally impractical and too expensive to perform AIMD simulations since the number of atoms (N) in the system exceeds 400 and the computational cost scales as ~$O(N^3)$. However, the energies obtained from the 1x1x4 grid are close to those from the finer grids. We also note that the value used for the cut-off energy (400 eV) is right on the border of where the plot in Supplementary Figure 13b starts converging. Using 400 eV, the error in the energy from its converged value of -3963.3562 eV is 0.07%, which makes it a reasonable balance between precision and computing cost.

Supplementary Note 5: Conformational changes of n-hexane in MD simulation

OPLS-AA potential[7] is used to model n-hexane and cyclohexane, which determines the bond lengths, angles and dihedrals of these molecules. This potential allows for small deformations about the mean for bond lengths, angles and dihedrals. However, completely relaxing angle parameters (while keeping the bond and dihedral parameters the same as the OPLS-AA model), achieved by setting the force constants (k_θ) of all angles in n-hexane and cyclohexane to zero, allows for large conformational changes in these molecules, thereby allowing them to freely stretch

and compress. Only after relaxing the angle parameters, we observed that n-hexane molecules could enter (6,5)-SWCNT, with cyclohexane still being excluded and this result is consistent with AIMD calculations. To estimate the threshold at which the hexane enters whereas cyclohexane cannot, a range of SWCNTs in increasing order of diameter, i.e., (6,5)-, (8,3)-, (7,5)-, (8,4)-, (7,6)- and (9,4)-SWCNT ((van der Waals pore size ranging from 0.422 nm to 0.581 nm) were investigated. We then kept the SWCNT in an n-hexane/cyclohexane bath at 300 K, as shown in Supplementary Figure 9, and modeled the system as an isothermal-isobaric ensemble, in which the number of molecules, pressure, and temperature were held constant. The MD results corroborate our experimental observations that (8,3)-SWCNT and large diameter nanotubes exhibit no selectivity (both n-hexane and cyclohexane are observed to enter the SWCNT (Supplementary Figure 14 and Supplementary Movies 3-6) while (6,5)-SWCNT exhibits selectivity allowing only n-hexane to enter the nanopore.

SWCNT chirality	carbon center-to-center diameter (nm)[8]	van der Waals pore size (nm)*
(6,5)	0.757	0.422
(8,3)	0.782	0.447
(7,5)	0.829	0.494
(8,4)	0.840	0.505
(7,6)	0.895	0.560
(9,4)	0.916	0.581

Supplementary Table 1. van der Waals pore sizes of the nanotubes used

* calculated by subtracting the van der Waals diameter of carbon atom from the carbon center-to-center diameter of SWCNTs. Note that the van der Waals diameter of carbon atom used here is the interlayer spacing in graphite (0.335 nm). The diameter of (*n*,*m*)-SWCNT is calculated from $d_t = \frac{\sqrt{3} l_{C\text{-}C}}{\pi} \sqrt{n^2+m^2+mn}$ based on wrapping a graphene sheet with a C-C bond distance $l_{C\text{-}C}$ of 1.44 Å.

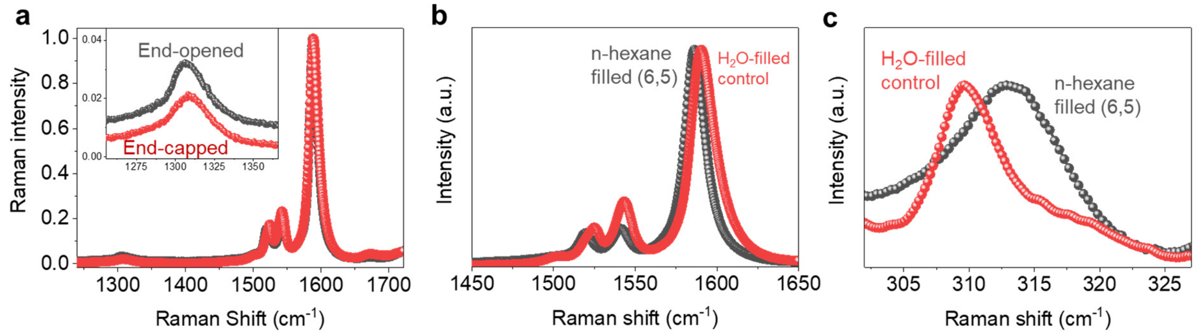

Supplementary Figure 1. Raman spectra of n-hexane-filled (6,5)-SWCNTs in comparison with H_2O-filled and end-capped controls. a, Raman spectra for end-opened SWCNTs (black curve) and end-capped SWCNTs (red curve). **b,** Raman spectra of (6,5)-enriched SWCNTs that are filled with n-hexane (black curve) and H_2O (red curve). **c,** RBMs of the n-hexane-filled and H_2O-filled (6,5)-SWCNTs. Note that all the spectra are normalized to the G or RBM peak intensity. Note: Raman spectra were collected under 532 nm laser excitation.

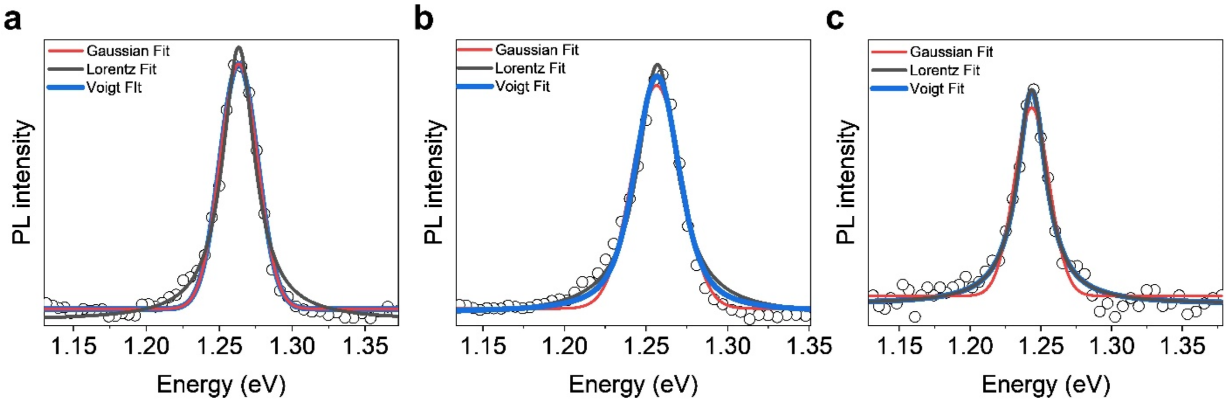

Supplementary Figure 2. Hyperspectral PL imaging spectra of individual (6,5)-SWCNTs fitted with Gaussian, Lorentzian and Voigt functions. a, Empty (6,5)-SWCNTs fitted with Gaussian, Lorentzian and Voigt functions. The peak positions from these fittings are all at 1.263 eV. **b,** Water-filled (6,5)-SWCNTs fitted with Gaussian, Lorentzian and Voigt functions. The peak positions from these fittings are all at 1.256 eV. **c,** n-hexane-filled (6,5)-SWCNTs fitted with Gaussian, Lorentzian and Voigt functions. The peak positions from these fittings are all at 1.243 eV. These results show that the peak position is not affect by the fitting functions, although their line widths do.

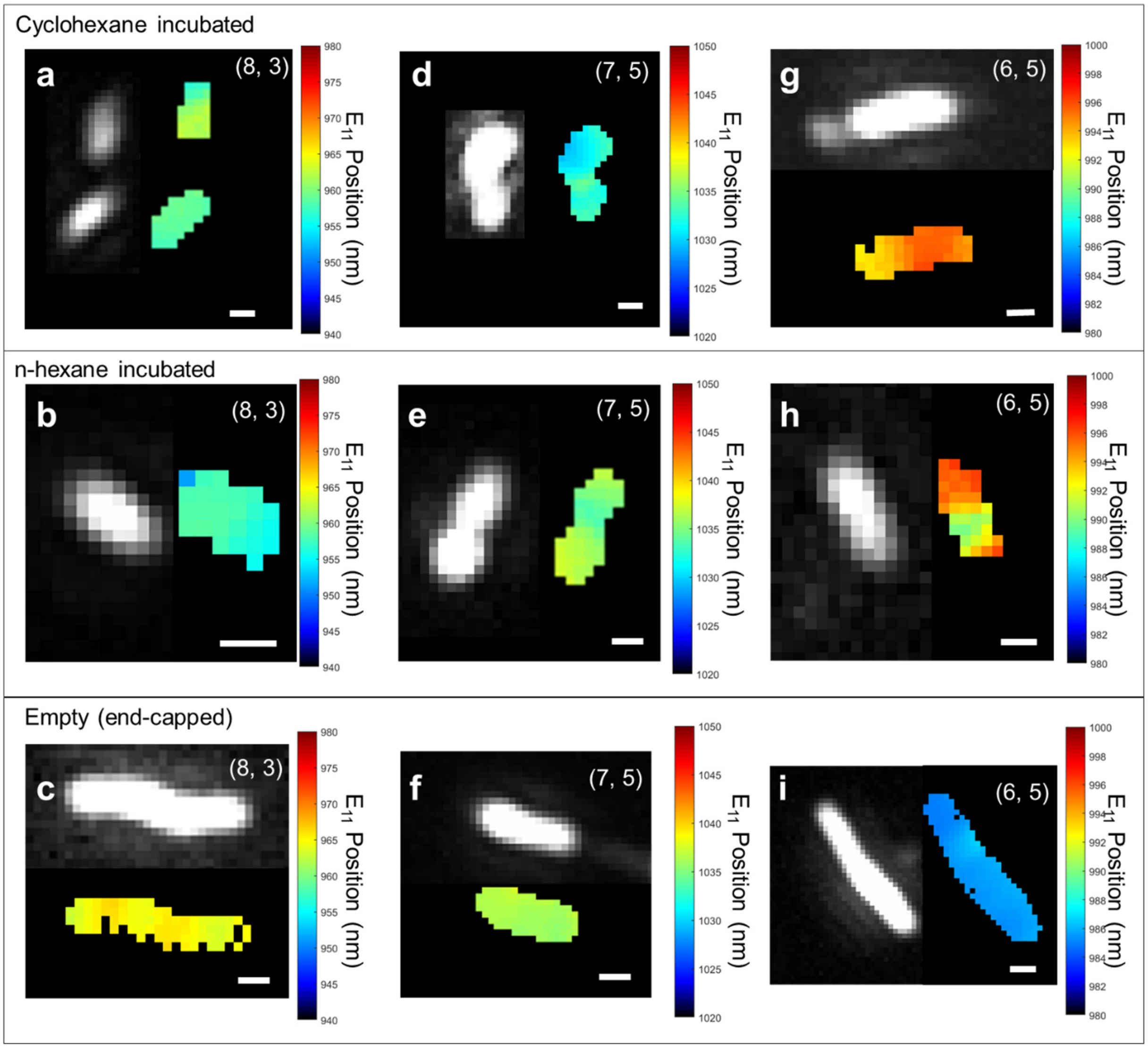

Supplementary Figure 3. Hyperspectral maps of individual SWCNTs that were incubated with cyclohexane, n-hexane, or left empty. a, A cyclohexane-incubated (8,3)-SWCNT. **b**, An n-hexane-incubated (8,3)-SWCNT. **c**, An empty (8,3)-SWCNT. **d**, A cyclohexane-incubated (7,5)-SWCNT. **e**, An n-hexane-incubated (7,5)-SWCNT. **f**, An empty (7,5)-SWCNT. **g**, A cyclohexane-incubated (6,5)-SWCNT. **h**, An n-hexane-incubated (6,5)-SWCNT. **i**, An empty (6,5)-SWCNT. For each nanotube, the PL peak position is plotted as a false color image along with the intensity image. Scale bars represent 500 nm. Note that the peak position is from spectral fitting of each data set with a Gaussian function.

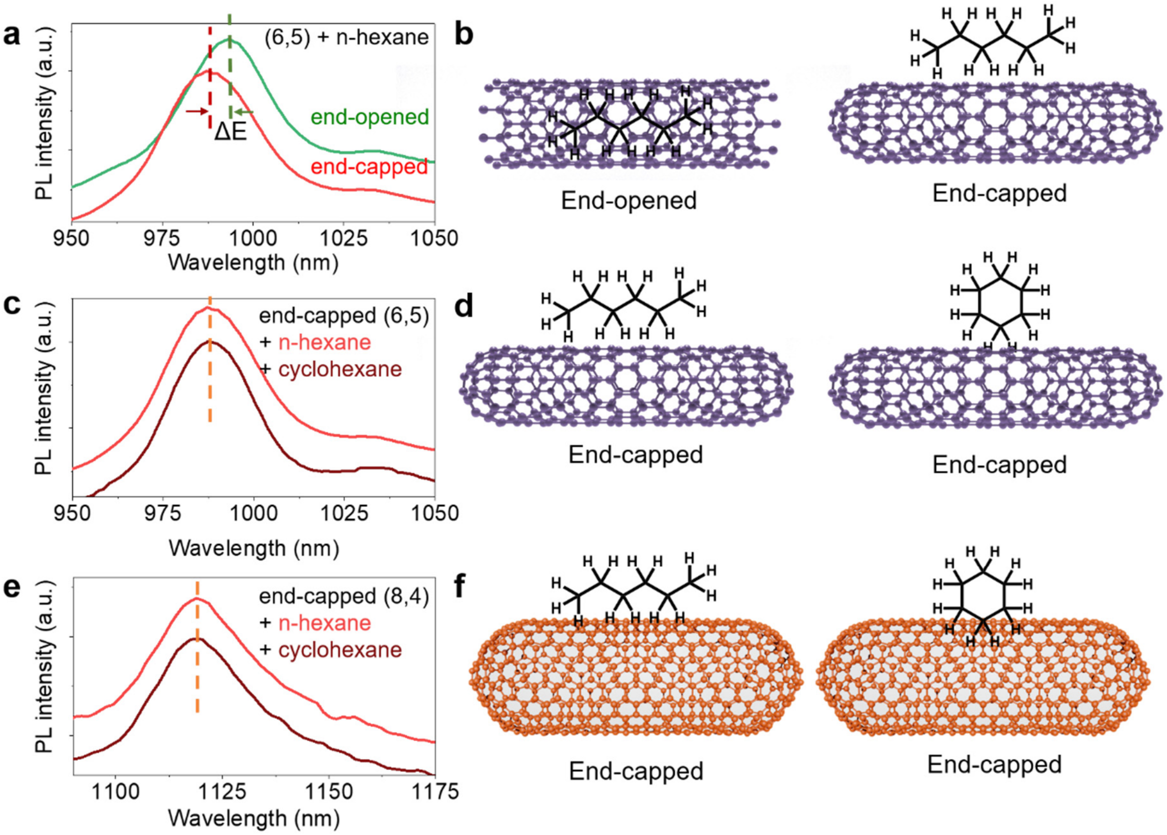

Supplementary Figure 4. Ensemble PL spectra of end-capped and end-opened SWCNTs that were incubated with n-hexane or cyclohexane. **a**, PL spectra and **b**, schematic of end-opened (green) and end-capped (6,5)-SWCNTs (red) that were incubated with n-hexane. **c**, PL spectra and **d**, schematic of end-capped (6,5)-SWCNTs that were incubated with n-hexane (light red) and cyclohexane (dark red). **e**, PL spectra and **f**, schematic of end-capped (8,4)-SWCNTs that were incubated in n-hexane (light red) and cyclohexane (dark red). Note that the spectra are normalized at the peak intensity and offset for clarity.

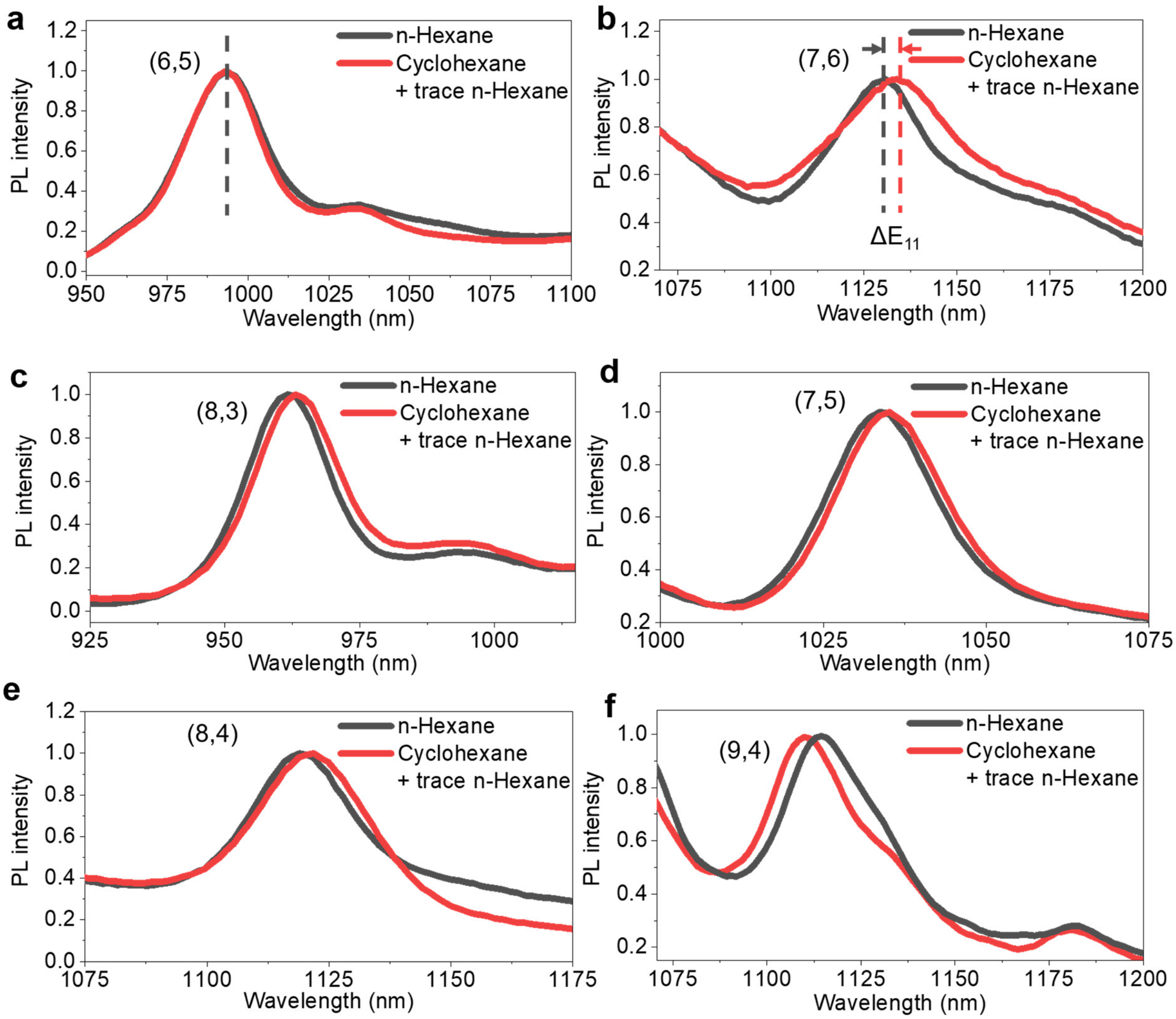

Supplementary Figure 5. End-opened Nanotube photoluminescence responds to filling molecules. **a**, (6,5); **b**, (7,6); **c**, (8,3); **d**, (7,5); **e**, (8,4); and **f**, (9,4) SWCNTs respond to cyclohexane (red curves) and n-hexane (black curves) with a spectral shift. The nanotube ends were opened prior to incubation with the solvents.

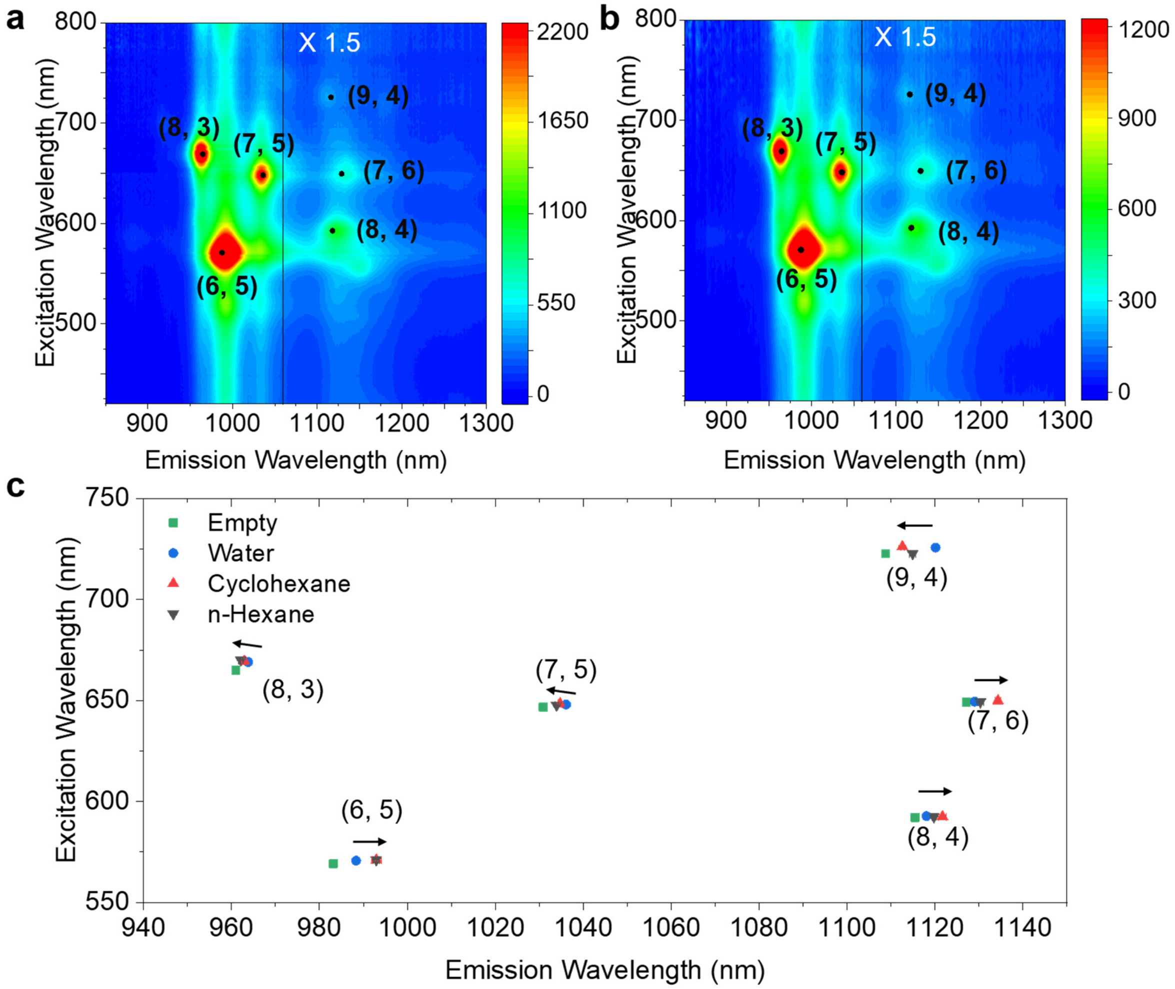

Supplementary Figure 6. Response of nanotube photoluminescence to filling molecules. Excitation-emission PL maps of SWCNTs that are incubated with **a**, cyclohexane and **b**, n-hexane. Note that the black dots mark the PL peak position of water-filled SWCNTs. The PL intensity beyond 1060 nm is plotted at 1.5× scale to make the minority nanotube species visible. **c**, Peak position of the E_{11} PL for SWCNTs incubated with cyclohexane (red) or n-hexane (black), and those filled with water (blue) or empty (green). The arrows indicate the direction of the spectral shift between water and alkane-filled species.

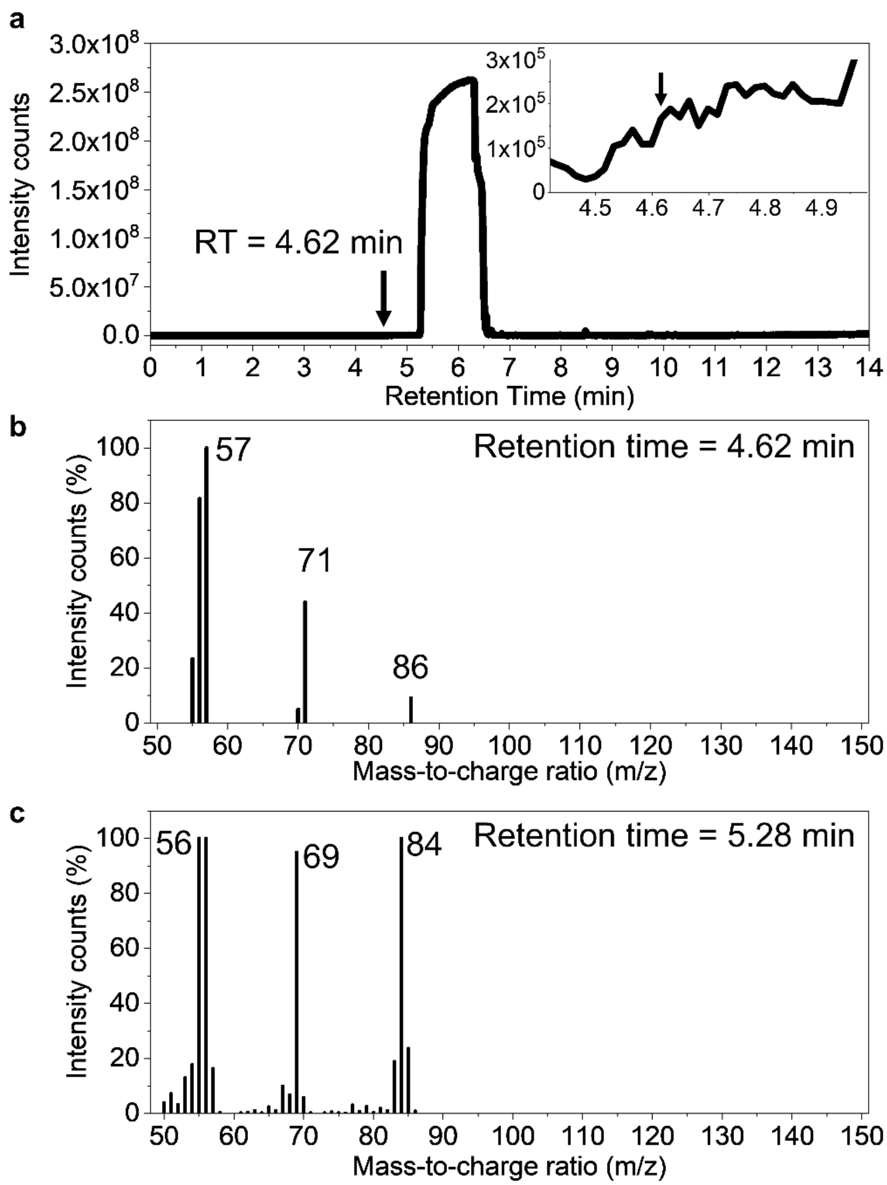

Supplementary Figure 7. GC-MS analysis of a commercial cyclohexane solvent (purity ≥ 99.90 %). a, Gas chromatogram of the cyclohexane. **b**, Mass spectrum of the component eluted at 4.62 min, which is assigned to n-hexane. Although there is no obvious peak in the gas chromatogram, the trace amount of n-hexane can be detected by the coupled JEOL high resolution magnetic sector mass spectrometer with an EI ion source (the detection sensitivity is on the order as low as 1 femtogram). **c**, Mass spectrum of the component eluted at 5.28 min, which is assigned to the majority cyclohexane component.

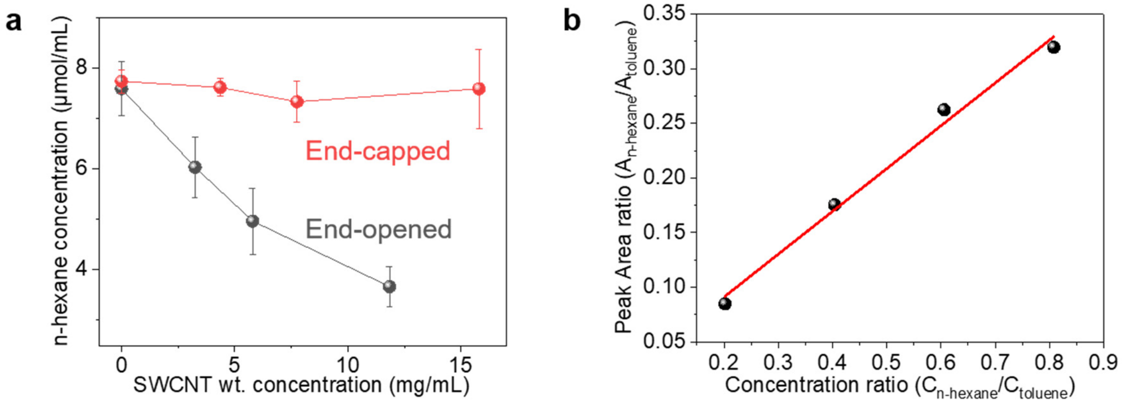

Supplementary Figure 8. GC-MS experiment for n-hexane concentration measurement. a, Selective adsorption of n-hexane comparison between end-closed and uncapped nanotubes. **b**, GC-MS calibration curve of concentration. The peak area ratio of hexane and toluene ($A_{\text{n-hexane}}/A_{\text{toluene}}$) is plotted as a function of the concentration ratio ($C_{\text{n-hexane}}/C_{\text{toluene}}$). The slope of the fitting curve is the response factor, which is ≈ 0.3918. $R^2 = 0.99$. Uncertainty in the calculated points are represented by error bars equal to one standard deviation.

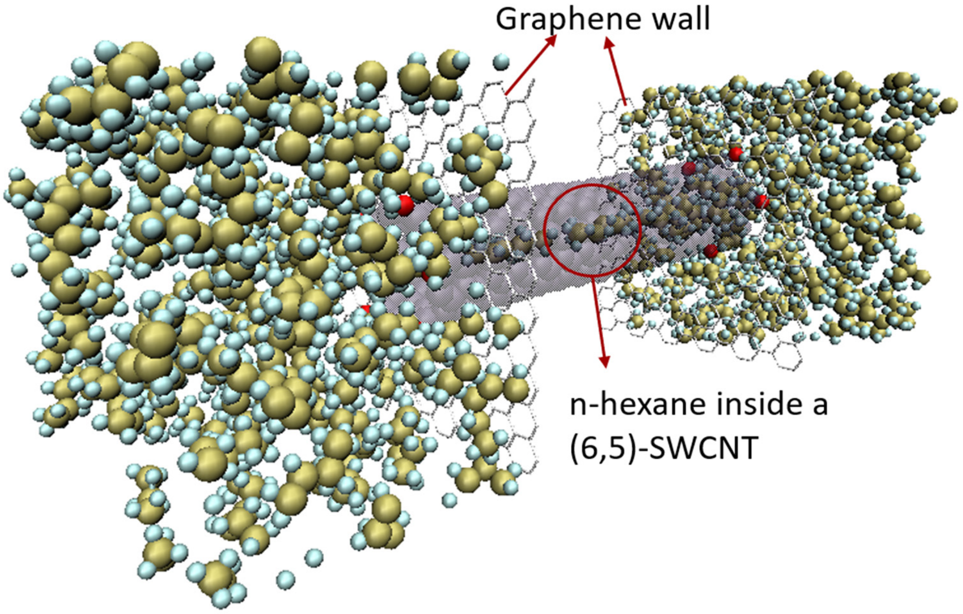

Supplementary Figure 9. The system used in the MD simulations, with SWCNTs emptying into cyclohexane (or n-hexane) baths kept at 300 K and 1 atm pressure. The ends of the SWCNT are terminated with hydroxyl (-OH) groups. Note that completely relaxed angle parameters are used for n-hexane in this simulation. The dimension of the cyclohexane (or n-hexane) bath is 2.5×2.5×2.5 nm^3, and the length of the SWCNT is 4 nm.

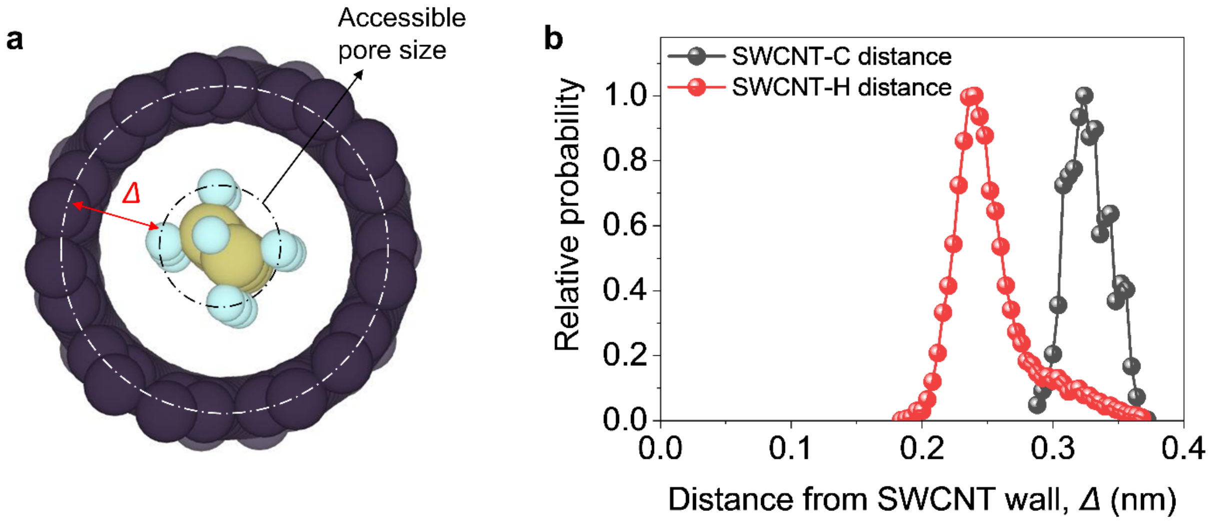

***Supplementary Figure 10. Accessible pore size from AIMD simulations. a**, Schematic of the accessible pore size, which is defined as the nanotube diameter subtracting the minimal atom-center-to-center distance between the (6,5)-SWCNT wall and n-hexane hydrogens. **b**, Probability distribution of the projected radial distance (Δ) between the carbon and hydrogen atoms of the n-hexane molecule and the (6,5)- SWCNT wall.*

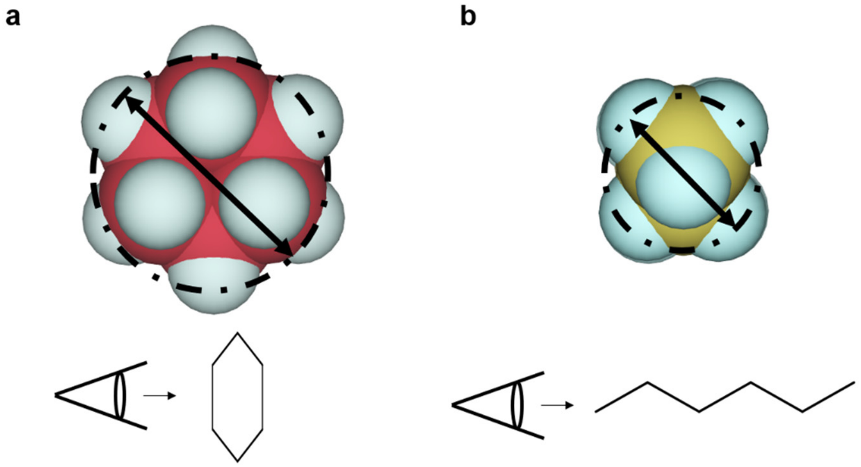

Supplementary Figure 11. Schematic showing the minimal projected diameter of a molecule. **a**, cyclohexane. **b**, n-hexane. Note, the projected diameter calculated in our simulations are atom center-to-center distance. Geometries for n-hexane and cyclohexane are optimized using Density Functional Theory (DFT).

	C-C bond length (Å)	C-H bond length (Å)	C-C-C bond angle	C-C-H bond angle	H-C-H bond angle	length of n-hexane (Å)*	minimum projected diameter (Å)
un-stretched	1.52	1.09	114.287˚	106.657˚	109.833˚	8.084	2.80
stretched	1.52	1.09	144.723˚	101.165˚	115.244˚	9.045	2.510

Supplementary Table 2. The molecular configurations of n-hexane under the confinement of (6,5)-SWCNT (stretched) and in the bulk solution (un-stretched) at 300 K and 1 atm. These values are calculated by averaging the respective bond lengths and angles for one sample configuration of stretched and un-stretched n-hexane (provided in the supplementary file). The sample configuration for stretched n-hexane is obtained when n-hexane is under confinement of (6,5)-SWCNT and for un-stretched n-hexane is obtained from the most probable configuration of n-hexane in the bulk solution at 300 K, 1 atm. These values are obtained from MD with systems described in the Methods section.

*Lengths of n-hexane molecules are end-to-end hydrogen distances.

Atom pairs	σ (Å)	ε (eV)
C-C	3.4	0.00286
C-O	3.024	0.0049
C-H	2.9	0.00192
O-H	3.166	0.00667
O-O	3.166	0.00667
H-H	2.4	0.00129

Supplementary Table 3. Lennard-Jones parameters used in MD simulations.

Grid size	Total electronic energy (eV)
1x1x4	-3963.3504 eV
2x2x2	-3963.3556 eV
4x4x1	-3963.3554 eV
4x4x4	-3963.3562 eV

Supplementary Table 4. Grid size and corresponding total electronic energy (eV) in AIMD

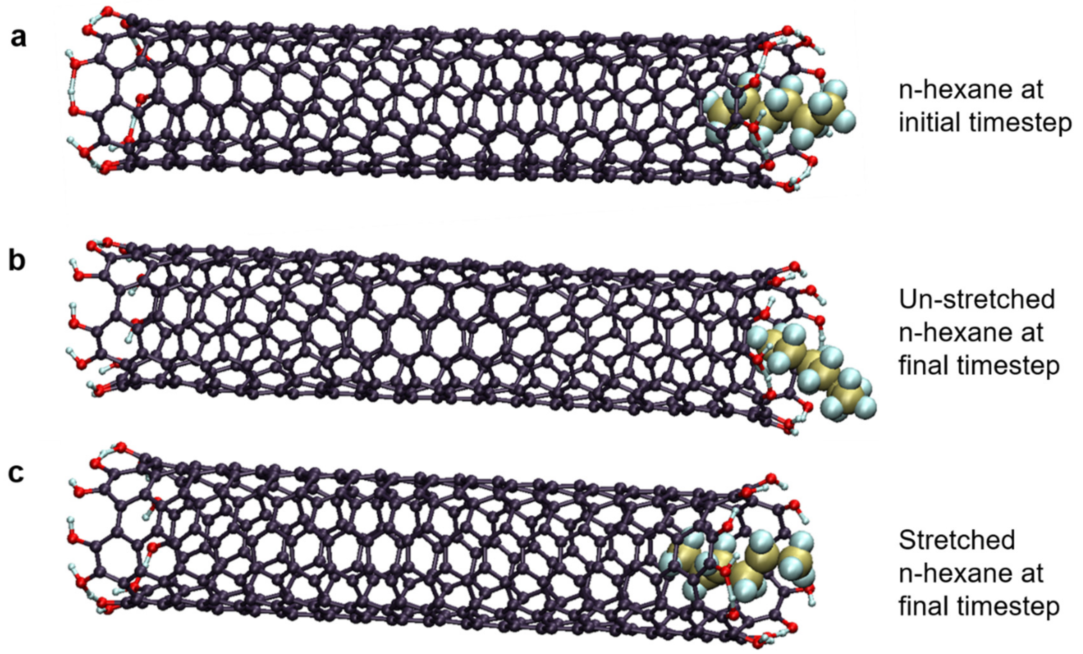

Supplementary Figure 12. AIMD simulations of filling a (6, 5)-SWCNT with stretched and un-stretched n-hexane. **a**, The n-hexane molecule is placed in the pore mouth at t = 0. **b**, The final configuration of un-stretched n-hexane at the end of the simulation at t = 2 ps (Supplementary movie 1). **c**, The final configuration of the stretched n-hexane at the end of the simulation at t = 2 ps. The AIMD simulations were performed at 300 K (Supplementary movie 2).

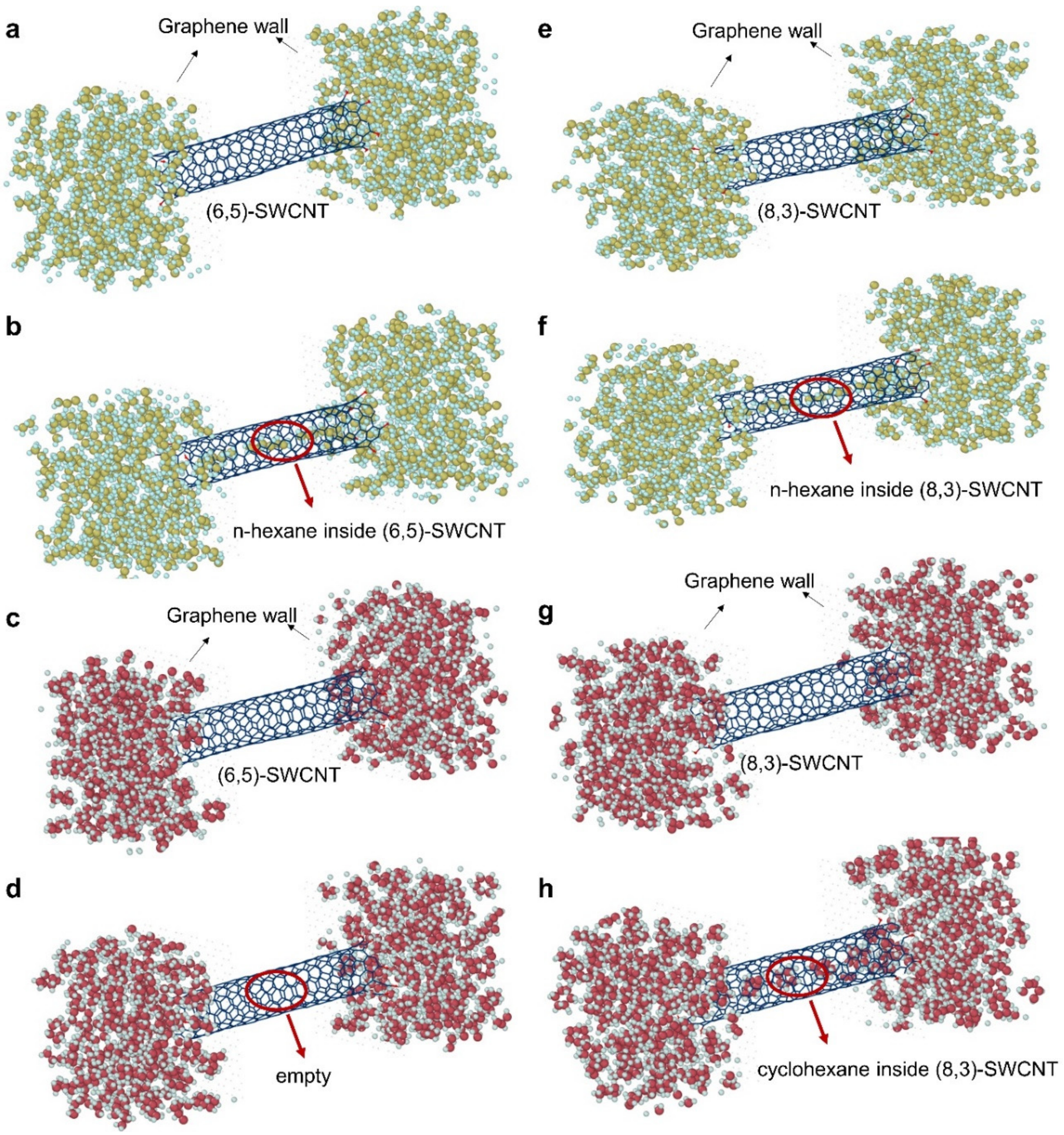

Supplementary Figure 13. MD simulations at 300 K, 1 atm of (6,5)- and (8,3)-SWCNT incubated with n-hexane or cyclohexane. a, A (6,5)-SWCNT placed in an n-hexane bath at t=0. **b**, Single file of n-hexane molecules filling in the pore of (6,5)-SWCNT at the final step t ≈ 200 ps (Supplementary movie 3). **c**, A (6,5)-SWCNT placed in a cyclohexane bath at t=0. **d**, Empty (6,5)-SWCNT at the final step t ≈ 200 ps (Supplementary movie 4). **e,** A (8,3)-SWCNT placed in an n-hexane bath at t=0. **f**, Single file of n-hexane molecules filling the (8,3)-SWCNT at the final step t ≈ 200 ps (Supplementary movie 5). **g**, A (8,3)-SWCNT placed in a cyclohexane bath at t=0. **h**, Single file of cyclohexane molecules filling the (8,3)-SWCNT at the final step t ≈ 200 ps (Supplementary movie 6). In all these snapshots, carbon atoms of n-hexane are depicted in yellow, carbon of cyclohexane depicted in maroon, hydrogen in light blue, (6,5)- and (8,3)-SWCNTs in dark blue. Note that the dimension of the cyclohexane (or n-hexane) bath is 2.5×2.5×2.5 nm^3, and the length of the SWCNT is 4 nm.

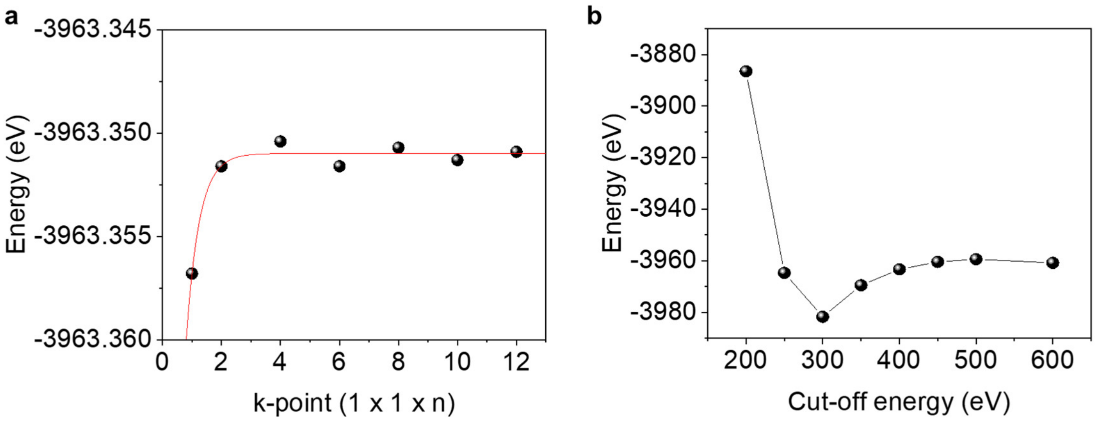

Supplementary Figure 14. Convergence from AIMD. a, Convergence as a function of grid size. **b,** Convergence as a function of cut-off energy

Supplementary References

1 Yang, L. & Han, J. Electronic structure of deformed carbon nanotubes. *Phys. Rev. Lett.* **85**, 154-157 (2000).

2 Okubo, S. *et al.* Diameter-Dependent Band Gap Modification of Single-Walled Carbon Nanotubes by Encapsulated Fullerenes. *J. Phys. Chem. C* **113**, 571-575 (2008).

3 Leeuw, T. K. *et al.* Strain measurements on individual single-walled carbon nanotubes in a polymer host: structure-dependent spectral shifts and load transfer. *Nano Lett.* **8**, 826-831 (2008).

4 Cambre, S. *et al.* Luminescence properties of individual empty and water-filled single-walled carbon nanotubes. *ACS Nano* **6**, 2649-2655 (2012).

5 Lee, A. J. *et al.* Bright Fluorescence from Individual Single-Walled Carbon Nanotubes. *Nano Lett.* **11**, 1636-1640 (2011).

6 Streit, J. *et al.* Alkane encapsulation induces strain in small-diameter single-wall carbon nanotubes. *J. Phys. Chem. C* **122**, 11577-11585 (2018).

7 Jorgensen, W. L., Maxwell, D. S. & Tirado-Rives, J. Development and Testing of the OPLS All-Atom Force Field on Conformational Energetics and Properties of Organic Liquids. *J. Am. Chem. Soc.* **118**, 11225-11236 (1996).

8 Dresselhaus, G., Dresselhaus, M. S. & Saito, R. *Physical properties of carbon nanotubes*. (World scientific, 1998).
