# Supplementary material for: Selective filling of n-hexane in a tight nanopore
Source: Nat Commun. 2021 Jan 12;12:310. doi: 10.1038/s41467-020-20587-1 (PMC7804426; doi:10.1038/s41467-020-20587-1)
Supplement: Supplementary file 3 — Description of Additional Supplementary Files [file 41467_2020_20587_MOESM3_ESM.docx]

**Description of Additional Supplementary Files**

**File:** Supplementary Data 1

**Description:** Geometry file of stretched n-hexane used in ab initio Molecular Dynamics simulation

**File:** Supplementary Data 2

**Description:** Geometry file of unstretched n-hexane used in ab initio Molecular Dynamics simulation

**File:** Supplementary Data 3

**Description:** Geometry file of (6,5)-SWCNT and unstretched n-hexane used in ab initio Molecular Dynamics simulation

**File:** Supplementary Data 4

**Description:** Geometry file of (6,5)-SWCNT and stretched n-hexane used in ab initio Molecular Dynamics simulation

**File:** Supplementary Movie 1

**Description:** movie of an unstretched n-hexane being excluded from a (6,5)-SWCNT by AIMD. The full length of the simulation is ≈ 2 ps.

**File:** Supplementary Movie 2

**Description:** movie of a stretched n-hexane entering a (6,5)-SWCNT by AIMD. The full length of the simulation is ≈ 2 ps.

**File:** Supplementary Movie 3

**Description:** movie of n-hexane entering a (6,5)-SWCNT. The full length of the simulation is ≈ 200 ps.

**File:** Supplementary Movie 4

**Description:** movie showing that cyclohexane cannot enter a (6,5)-SWCNT. The full length of the simulation is ≈ 200 ps.

**File:** Supplementary Movie 5

**Description:** movie of n-hexane entering a (8,3)-SWCNT. The full length of the simulation is ≈ 200 ps.

**File:** Supplementary Movie 6

**Description:** movie of cyclohexane entering a (8,3)-SWCNT. The full length of the simulation is ≈ 200 ps.
